# An Old Man With Acute Visual Loss

**DOI:** 10.1016/j.acepjo.2025.100148

**Published:** 2025-05-05

**Authors:** Tse-Ying Lee, Wan-Ching Lien

**Affiliations:** 1 Department of Emergency Medicine, National Taiwan University Hospital, Taipei, Taiwan; 2 Department of Emergency Medicine, College of Medicine, National Taiwan University, Taipei, Taiwan

**Keywords:** painless visual loss, submacular hemorrhage, point-of-care ultrasound

## 1 Patient Presentation

A 78-year-old man with no significant medical history presented with a 1-day history of painless visual loss, described as a blackish area in the nasal field of his left eye. Examination revealed no eyelid or conjunctival abnormalities. Because of the unavailability of an ophthalmologist, an ocular point-of-care ultrasound was performed (Fig, A).

**Figure:**
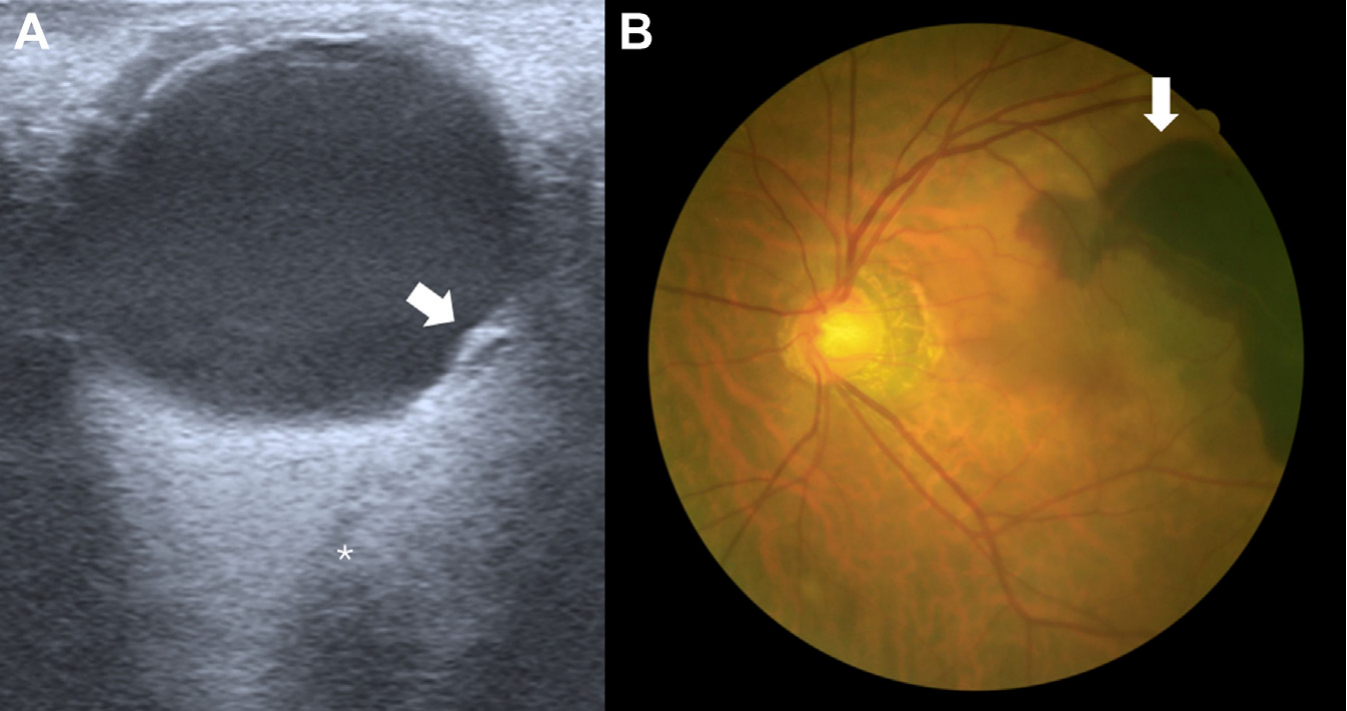
(A) The ocular ultrasound showed a bulging retina (arrow) with a mixed echogenic material posterior the lesion. ∗Indicates the optic nerve. (B) The fundus photography showed an irregularly shaped area of black discoloration (arrow) beneath the retina.

## 2 Diagnosis: Submacular Hemorrhage

The diagnosis was later confirmed by fundus photography (Fig, B). The patient received 2 intravitreal bevacizumab injections but developed a massive macular and vitreous hemorrhage, leading to a significant visual decline. He was subsequently lost to follow-up.

Common causes of acute painless visual loss include retinal detachment, vitreous hemorrhage, and posterior vitreous detachment.^1^ Point-of-care ultrasound aids in emergency settings when immediate ophthalmologic examination is unavailable. Vitreous hemorrhage appears as multiple dot-like echoes, retinal detachment as a uniformly thick hyperechoic line, and posterior vitreous detachment as a more mobile hyperechoic line crossing the optic nerve.

Submacular hemorrhage, a rare cause of acute painless visual loss, involves blood accumulation between the retinal pigment epithelium and neurosensory retina, often linked to choroidal neovascularization in age-related macular degeneration.^2^ Submacular hemorrhage presents as a bulging retina with mixed echogenic material posterior to the lesion, as seen in this case.

## Funding and Support

10.13039/100020595National Science and Technology Council, Taiwan (NSTC 113-2410-H-002 -166 -MY2).
